# Three contextual cues and their influence on naming in children

**DOI:** 10.1002/jeab.70059

**Published:** 2025-10-10

**Authors:** Nadine Hempkin, Maithri Sivaraman, Dermot Barnes‐Holmes

**Affiliations:** ^1^ Mohammed Bin Rashid Center for Special Education, Operated by the New England Center for Children Abu Dhabi United Arab Emirates; ^2^ School of Psychology Ulster University Coleraine Northern Ireland; ^3^ Teachers College Columbia University USA

**Keywords:** autism, children, contextual cues, incidental naming

## Abstract

Children often learn the names of objects incidentally—that is without direct instruction or programmed reinforcement—simply by observing others label novel objects. A number of contextual cues have been deemed important in the development of naming such as orientation toward stimuli, pointing, linguistic prompts (e.g., “This is…”), and contiguous presentations of stimuli and sounds. Despite their significance, there has been almost no systematic investigation of these cues in behavior analysis. The current study preliminarily examines how contextual cues—such as an experimenter's eye gaze, pointing, and use of paralinguistic cues—affect naming responses. In Experiment 1, three typically developing children were administered naming tests with and without these cues using a reversal design. All participants showed improved performance with cues relative to without cues. Experiment 2 extended this by testing three autistic children with all cues, a partial set of cues, or no cues, using a reversal design. Results replicated Experiment 1, also demonstrating that partial cues were effective in facilitating naming. Experiment 3 replicated the results of Experiment 1 with three additional autistic participants during which test trials remained consistent across conditions in a reversal design. Further research on the contextual cues presented during naming experiences is warranted.

*Naming* was initially described by Horne and Lowe (1996) as a verbal developmental stage. Since their seminal article, multiple taxonomies have been proposed to describe the subtypes of naming (e.g., see Hawkins et al., 2018; Miguel, 2016). One subtype, called incidental naming, has been described as children learning the names of objects through *incidental exposure*—that is, without direct instruction or reinforcement. Rather, a child learns to relate the name of the stimulus to the actual stimulus, as a listener and/or a speaker, through repeated “pairings” of names and stimuli. Such events have been described as naming experiences (Greer & Longano, 2010). Commonly used in the literature and adopted by the current article, have been the terms listener and speaker naming to differentiate between topographies of naming responses. Speaker naming (Greer et al., 2005) describes a child responding to the environment as a speaker following a naming experience. For example, hearing someone label a “book” as the child and speaker both observe the book may subsequently result in the child saying the word “book” in its presence. Listener naming refers to a child responding to stimuli as a listener simply after observing others label a novel stimulus (Greer et al., 2011). For example, when hearing the word “book,” a young child may point or orient toward the book.

The establishment of speaker and listener naming has been widely studied in both typically developing and autistic children (e.g., Gilic & Greer, 2011; Greer et al., 2005, 2011; Miguel & Kobari‐Wright, 2013). However, a recent review by Sivaraman and Barnes‐Holmes (2023) found wide variation in the methods used to test and train naming. Specifically, the review revealed differences in the types of stimuli used, the naming experiences used to pair spoken names with stimuli, the number of novel stimuli presented to a child during a test, and the number and types of trials used to test for naming.

Sivaraman and Barnes‐Holmes (2023) also highlighted that only two of the included studies involved conditions in which the object and its name were not presented simultaneously. It can be argued that when a name and an object are presented together, the bidirectional relation between the name and the object can be *directly* established. That is, when the object and word are presented together, the child may effectively “hear‐name and see‐object” and also “see‐object and hear‐name.” On the other hand, imagine that an object and name are presented nonsimultaneously (i.e., present the object, hide the object from view and then state the name of the object; *object–name* relation). In the natural environment, this might occur, for example, when a family is driving and a parent and child both observe a bird flying past. Moments later, when the bird is no longer in view, the parent might ask, “Did you see the eagle?” and point in the general direction where the bird was seen. In this case, a symmetrical or mutually entailed derived *name–object* relational response may be involved during a subsequent test for naming (i.e., when the child is asked to name an eagle the next time they see one). In other words, the *name–object* relation is derived from the directly experienced *object–name* relation because they were not presented simultaneously. As an aside, the explanation for this derivation and the naming responses remains a matter of debate (e.g., mediational events, such as private tacts and echoics, and/or multiple sources of contextual control established by a history of appropriate multiple exemplars; see Regaço et al., 2025).

In studying the emergence of speaker and listener naming, there is a lack of research on the influence of inserting delays between object and name presentations.^1^ However, recent research has begun to explore the influence of such delays and demonstrated improvements in speaker and listener naming in young children following multiple‐exemplar training (MET) when objects and names are presented nonsimultaneously (e.g., Sivaraman et al., 2021). These researchers found that MET induced generalized listener responses in all participants and speaker responses in one participant.

In focusing on the influence of object–name delays on children's ability to learn new names, it is important to ask why such delays may have a negative effect on naming responses. In other words, the simultaneous presentation of a name and its corresponding object (as opposed to a nonsimultaneous arrangement) appears to facilitate the learning of names. According to relational frame theory (RFT), derived relational responses are under the control of specific contextual cues and thus, from this theoretical perspective, derived naming likely involves some form of contextual control. According to RFT, contextual cues in a naming experience (i.e., the linguistic cues such as “This is…” and paralinguistic cues such as pointing and holding up the stimulus, looking back‐and‐forth between the child and the object) may serve as stimuli that establish the relation between an object and its name (see Gilmore et al., 2024, for a detailed overview). For young children, all these cues are potentially critical in establishing naming responses. Interestingly, many of the studies reported in the Sivaraman and Barnes‐Holmes (2023) review involved researchers pointing to the stimulus or offering other paralinguistic cues *simultaneously* during sound‐stimulus exposure trials. However, the specific effects of these cues has never been studied systematically, and it is important to do so to deepen understanding of the cues that facilitate naming in young children.

Although research on the effects of these linguistic and paralinguistic cues has not been conducted within behavior analysis (but see Olaff & Holth, 2020), researchers in allied disciplines have pursued scientific enquiry on this topic. For instance, multiple longitudinal research studies (e.g., Carpenter et al., 1998; Mundy et al., 2007) have shown that early word learning, both as a speaker and as a listener, is positively correlated with joint attention and orienting toward stimuli. In other studies (e.g., Slaughter & McConnell, 2003; Tenenbaum et al., 2014), the researchers found that infants' learning of specific names for objects was positively influenced by following an adult's gaze or following an adult pointing toward a novel object. For example, Striano et al. (2006) exposed infants to two conditions, a *joint attention* condition in which the experimenter alternated their gaze from the object to the infant while stating the object's name and an *object‐only* condition in which the experimenter gazed at the object and at a spot on the ceiling while stating the object's name. They found that 9‐month‐old participants gazed longer at the novel object than at the familiar object following the joint‐attention condition than following the object‐only condition. Overall, prior research in developmental and experimental psychology has exposed children to a range of social cues (e.g., eye gaze directed at the child, pointing) and found that these cues facilitate children's responses to novel objects/words (see Lee & Lew‐Williams, 2022, for a recent review). Given these findings, it seems important to explore the role played by various contextual cues, such as pointing and looking at the named object and simultaneously uttering vocal cues, such as “This is a…” or “Can you see the …,” in learning to name.

A parent's use of pointing gestures has also been shown to be related more directly to infant language development (Rowe, 2000). When caregivers synchronize their gesture use with linguistic cues about an object or event, learning of novel words is facilitated more than when linguistic cues occur with no gestures or with asynchronized gestures (de Villiers‐Rader & Zukow‐Goldring, 2012). Despite the apparent importance of these paralinguistic cues, they have never been investigated systematically by behavior analysts in the context of incidental naming. At this point, therefore, it seems wise to operationalize and systematically manipulate these cues to investigate the role they may play in the development of more effective interventions in teaching and training naming abilities. For example, it remains to be determined which of these cues are necessary, whether their inclusion enhances the effectiveness of the naming experience, and whether their removal hinders the learning of new names.

Given the absence of behavior analytic research in the area, the current study focused on three contextual cues that potentially play an important role in children learning to name. Specifically, we investigated the influence of linguistic cues (e.g., “This is …”), pointing, and eye gaze on children's speaker and listener naming across three experiments in which novel objects and their names were presented nonsimultaneously. We tested both speaker and listener naming because both response topographies are required to demonstrate bidirectional naming (Greer et al., 2005). We chose these specific cues based on their prevalence in prior research (e.g., Rowe, 2000; Striano et al., 2006). Furthermore, naming tests in the current study were presented with one stimulus at a time to be consistent with the vast body of studies in developmental psychology that adopted the same approach (e.g., Akhtar et al., 2001; Fitch et al., 2020; Scofield et al., 2011). In Experiment 1, all three cues were presented and then removed during nonsimultaneous presentations of objects and their names with three typically developing children who showed evidence of naming abilities. Experiment 2 attempted to replicate the results of Experiment 1 and examined the influence of pointing in the absence of eye gaze and linguistic cues with autistic children. Experiment 3 attempted to replicate the results of Experiment 1, again with autistic children, but by maintaining consistency in presentation across test trials.

## EXPERIMENT 1

### Method

#### Participants

Participants were three typically developing toddlers. At the beginning of the study, P1 was a 3‐year‐and‐3‐month‐old female and P2 and P3 were both 3‐year‐and‐4‐month‐old males. All participants completed the study within 6 weeks of beginning the study. Participants were recruited from staff who worked at a local intensive behavioral intervention center. All participants had previously completed listener and speaker responses in a core skills assessment (a standardized assessment used by a local intensive behavioral intervention center to assess children's behavioral repertoires across multiple domains such as communication, self‐help skills, social skills, preacademic and academic skills, see Dickson et al. 2014, for more information). Based on the results of this assessment, all three participants were deemed to have strong preexisting English listener and speaker responses and other communicative skills such as joint attention. All participants were also reported to have listener and speaker responses by their caregivers. For example, the caregivers of all participants noted they could pick up/point to familiar objects when asked to do so, such as car, chair, ball, and bubbles, and could label familiar objects such as doll, dog, and head when shown a picture and asked, “What is this?” All participants were reported to be able to ask for their preferred food or activities (e.g., cars, dolls, ice cream, playground). All participants could speak in sentences and phrases.

The first author, who was a master's‐level behavior analyst with over 6.5 years of experience working with children, served as the experimenter for all three experiments. She was also a lead therapist who worked alongside other clinicians providing and supervising educational and behavioral services at the center.

Ethical approval for all experiments was obtained from the first author's educational institution where she is enrolled as a doctoral student and from the center in which she worked. Parents of the participants provided written informed consent, and verbal assent was sought from each participant prior to each session.

#### Setting and materials

All sessions took place in a research room in the center (7 × 8 m) equipped with two tables, one adult chair, and one child chair. The experimenter and the participant were present during the sessions. All sessions were videotaped for reliability purposes.

Materials included 15 different objects (average size 11 × 2 × 5 cm) and images (7 × 5 cm) and arbitrary spoken sounds, presented across five conditions (three objects/images per condition). All the visual stimuli used during training were unfamiliar to the participants (based on caregiver report) and all the spoken sounds were nonsense words. A sample of 10 stimuli is provided in Table 1. A complete list of all the stimuli used during the study is provided in the supplementary materials. The selection and order of the stimuli were randomly assigned to each participant across conditions. For all participants, objects were used, except for P2, where we used images instead of objects because he would often try to play with the objects and did not attend to the experimenter's instructions when the objects were around.

**TABLE 1:** Sample of stimuli used during training for all experiments.

Stimulus	Name	Picture
1	MESA	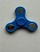
2	BOBO	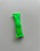
3	NAJ	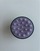
4	BEK	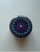
5	FED	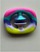
6	AHMIT	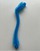
7	MOT	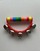
8	TIC	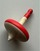
9	PAM	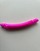
10	ADD	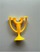

*Note*: Photos depict the physical objects used in the experiment. For P2, these photos were presented as stimuli in place of the physical objects.

#### Response measures, interobserver agreement, and procedural fidelity

The main dependent variable was percentage of correct responses. A correct response was defined as touching, pointing to, or picking up the correct comparison during listener trials, and saying the name of the stimulus during the speaker trials.

The first observer recorded data live during the sessions. A second independent observer recorded data from the video recordings for approximately 36% of listener and speaker trials during the test sessions for each participant. For each trial (during a test session), an agreement occurred if both observers scored either correct or incorrect and a disagreement occurred if one of the observers scored correct and the other scored incorrect. Overall agreement during test sessions was 100% for listener trials and 93.3% for speaker trials for P1. Overall agreement during test sessions was 93.3% for listener trials and 86.7% for speaker trials for P2. Overall agreement during test sessions 93.3% for listener trials was and 93.3% for speaker trials for P3.

We assessed procedural fidelity for approximately 30% of sessions. For each session, we measured whether each naming experience and listener and speaker trial was delivered with the correct/incorrect antecedent cues. Nine such measurements were made for each session and the overall percentage of procedural fidelity was calculated using the total number of correct presentations divided by the total number of presentations. Overall procedural fidelity for P1 was 96.3%, for P2 was 92.6%, and for P3 was 92.6%.

#### Procedure

All sessions began with the experimenter building rapport with the child. The experimenter played with the child for 2–5 min to establish rapport and encourage active responding. This involved the experimenter sitting facing the child across a table. The experimenter then presented the target novel object (e.g., BOBO) and two other novel objects (e.g., AHMIT, FED) to the child for a few seconds to remove novelty effects. The experimenter encouraged the child to look at and touch each object at least once. This was conducted for all objects.

##### Echoic pretest

We conducted this test prior to the commencement of the study. During this test, the experimenter stated each arbitrary spoken sound (see Table 1) with the instruction “Say [sound]” and noted whether the participant echoed the sound back to the experimenter. The purpose of this pretest was to determine whether the child had the motor responses to say the names chosen for the study. If the child was unable to say a given name, then it was not included in the study for that child, and a new sound that the child could produce was included instead. This ensured that the complexity of the vocal responses was consistent across all conditions. This test also affirmed that each child had the ability to echo responses. Therefore, any incorrect responses during the naming test would be attributed to being unable to name rather than being unable to produce the sound that was previously heard.

##### General procedure

Each session involved one or more test administrations within the same phase. When a phase change occurred (i.e., from the with‐cues to the without‐cues condition), we began a new session on a separate day. A parent accompanied the child to each session, but they sat outside the room. Sessions for all participants lasted on average between 50 and 70 min. It took five sessions in total for each participant, with P2 and P3 needing additional sessions for pairing and rapport building. During each session, the experimenter administered naming tests, which involved a stimulus and its name being presented to the child followed by a series of listener and speaker trials (all conducted nonsimultaneously, see more below). Each test was administered with a novel stimulus.

##### Naming test with‐cues condition

This test was conducted in the exact manner as Sivaraman et al. (2021) and is described below.

*Three object‐sound exposure trials*. The experimenter sat facing the child either across a table or on the floor. The experimenter held up an opaque box and said, “Wow, what is in here?” to keep the child motivated. The experimenter then removed an object from the box, asked the child to attend to the object (e.g., BOBO), which the experimenter held up in her hand, pointed to, shifted her gaze between child and object, and said, “Look at this.” When the child appeared to make visual contact with the object, the experimenter placed the object back in the box, then pointed to the box, gazed between the box and the child, and said, “This is [the name of the object]” (e.g., “This is BOBO”). That is, the presentation of the object and the name was nonsimultaneous. No specific consequences were administered. This trial was called an object‐sound exposure trial, and it was repeated two more times with the same object with 2‐ to 4‐min intertrial intervals. The only expectation of the child during these trials was that they appeared to look at the object (i.e., the child oriented their gaze toward the object for at least 2 s). For P2, these trials (and all subsequent trials) were conducted in the same manner as described with the box, but we used images instead of objects. Consistency in the delay between stimuli and their corresponding names was achieved by ensuring the participant was attending to the stimuli and not proceeding with the trial until attention was gained.

*Intertrial intervals*. During the intertrial intervals, the experimenter and the child engaged in unrelated play activities to maintain the child's motivation. Throughout these intervals, intermittent social praise was provided to the child for sitting, attending, and engaging in play with the experimenter; the child was also able to engage in preferred activities. Intertrial intervals ranged from 2 to 4 min. Variability in intertrial intervals may have been due to the child giving up the toys they were playing with or the researcher having to remove the toys. Additionally, sometimes participants would get up and walk around the room and time would be taken to encourage the child to come back or attend to the experimental task. Thus, there were small variations in intertrial intervals between participants depending on each child's motivation.

*Three speaker trials*. After a 2‐ to 4‐min intertrial interval following the third object‐sound exposure trial, three speaker trials were conducted. During the speaker trials, the experimenter held up the object (e.g., BOBO) and once the participant appeared to look at it, the experimenter placed it back in the box, pointed to the box, shifted her gaze between child and the box, and asked, “What is this?” The intertrial interval between the speaker trials was approximately 2–4 min.

*Three listener trials*. After a 2‐ to 4‐min intertrial interval following the third speaker trial, three listener trials were conducted. During these trials, the experimenter asked the child to point at or pick up the object previously named (e.g., “Point to/pick up/which one is [the name of the object]; e.g., “Point to/pick up/which one is BOBO.”). The experimenter then presented the object that was previously named (e.g., BOBO) with two other objects. Note that the name was presented first (i.e., “point to BOBO.”) and then the array of objects was made visible to the child. No differential consequences were provided for correct or incorrect responses. The intertrial interval between the listener trials was approximately 2–4 min.

Three such tests were conducted for each child during this phase. The tests were conducted in the exact manner as described above but we used novel stimuli from Table 1 each time.

##### Naming test without‐cues condition

*Three object‐sound exposure trials*. The only difference between the with‐cues and without‐cues condition was that in the without‐cues condition, no linguistic or paralinguistic cues were provided. Specifically, when visual contact occurred, the experimenter placed the object back in the box, left the box on the table, and said, “[the name of the object];” e.g., “BOBO”). The experimenter did not look at the object and kept her gaze focused away from the child and the box while saying the name of the object. In addition, the experimenter's hands remained on her lap away from the box. There were no other differences between the two conditions.

*Intertrial intervals*. These were the same as the intertrial intervals in the with‐cues condition.

*Three speaker trials*. These were conducted similarly to the with‐cues condition, except the experimenter did not look at the object/box and had her gaze focused away from the child and box while asking, “What is this?” The experimenter's hands were on her lap away from the box.

*Three listener trials*. These were conducted similarly to the with‐cues condition, except that the experimenter gazed away from the child and the box when presenting the instruction to “Pick up” or “Point to” the previously presented object. The experimenter gazed toward the participant once the participant selected an object from the array.

Similar to the with‐cues condition, during each phase of the without‐cues condition, we conducted three tests each with novel stimuli.

#### Experimental design and data analysis

An ABABA reversal design was used wherein A was the with‐cues condition and B was the without‐cues condition. During each A or B phase, three naming tests were administered as mentioned earlier. Supplementing the visual analysis, we conducted chi‐square tests to compare participants' responses during each phase.

### Results and discussion

**FIGURE 1:**
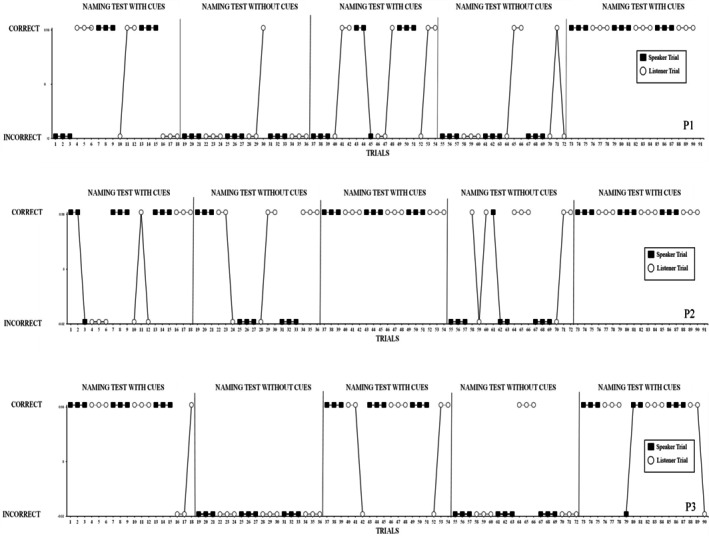
Responses of P1, P2, and P3 during administration of the naming test with and without cues.

The results of all three participants are displayed in Figure 1. Figure 1 displays P1, P2 and P3's results during administrations of the naming test with and without cues. P1 had 20/27 correct speaker responses and 19/27 correct listener responses during the with‐cues condition. Comparatively, P1 has 0/18 correct speaker responses and 4/18 correct listener responses in the without‐cues condition. Overall, P1 emitted more errors during the without‐cues condition than during the with‐cues condition. P2 produced 26/27 correct speaker responses and 22/27 correct listener responses during the with‐cues condition. Comparatively, P2 demonstrated 4/18 correct speaker responses and 14/18 correct listener responses during the without‐cues condition. Overall, P2 emitted substantially more errors during speaker trials in the without‐cues condition than in the with‐cues condition. P3 produced 26/27 correct speaker responses and 22/27 correct listener responses during the with‐cues condition. Comparatively, P3 has 0/18 correct speaker responses and 3/18 correct listener responses in the without‐cues condition. Overall, P3 emitted more errors during the without‐cues condition than during the with‐cues condition.

The results indicate clear differences in naming when tests were presented with and without cues, with all participants producing more correct responses during test conditions with cues than during those without cues. A Pearson's chi‐square test indicated that the total number of correct responses on listener and speaker trials was significantly associated with the presence or absence of cues, χ^2^_(1)_ = 97.22, *p* < .0001. More specifically, a participant was more likely to emit a correct speaker or listener response during trials where stimuli were presented with cues and less likely to emit a correct speaker or listener response during trials presented without cues.

Participants generally emitted no or relatively few correct speaker responses during the without‐cues condition than during the with‐cues condition. Furthermore, participants were more likely to emit correct listener responses during the without‐cues condition, but these were fewer than during the with‐cues condition (except P2 who produced similar levels of correct responding during both conditions). A Pearson's chi‐squared analysis indicated that responses during speaker trials were significantly associated with the presence or absence of cues, χ^2^_(1)_ = 87.43, *p* < .0001, and that responses during listener trials were also significantly associated with the presence or absence of cues, χ^2^_(1)_ = 21.90, *p* < .0001. Thus, although both speaker and listener responses were influenced by the presence or absence of cues, speaker responses appeared to be more so.

Experiment 1 investigated the influence of three contextual cues on typically developing young children's naming experiences. Overall, each participant emitted more correct naming responses during test conditions where stimuli were presented with cues than during test conditions where stimuli were presented without cues. These findings highlight the importance that contextual cues have during naming responses, particularly in young children. However, we did not test the individual influence of specific cues in this experiment.

Some autistic children demonstrate difficulties in the functional use of language relative to their typically developing peers (Anderson et al., 2007). Given that some of these children demonstrate difficulties in understanding the cues of others (e.g., Jellema et al., 2009), it seems worthwhile to further explore the specific influence of contextual cues (e.g., pointing, orienting, and linguistic cues) that may be incorporated into training and/or testing for naming. Therefore, Experiment 2 investigated the role of one such contextual cue in autistic children: pointing. Therefore, we systematically removed eye gaze and linguistic contextual cues but not pointing during a partial‐cues test condition to test the influence during a naming experience. Given the prior findings demonstrating the importance of pointing in early childhood (Bates et al.,^1975^; Bertenthal et al., 2014; Butterworth, 2003; Mundy et al., 2007), we chose to focus on this cue.

## EXPERIMENT 2

### Method

#### Participants

The participants were three autistic children. At the beginning of the study, P4 was a 7‐year‐old male, P5 was a 6‐year‐and‐5‐month‐old female, and P6 was a 7‐year‐and‐8‐month‐old male. All participants completed the study within 6 weeks of beginning the study. All participants had previously completed the same core skills assessment used in Experiment 1 and were found to have the same preexisting skills. However, these participants were noted to be unable to engage in extended conversations or interact with peers through play (e.g., games of cooperation, initiation of peer play).

#### Setting and materials

The setting was the same as for Experiment 1. All sessions were again videotaped for reliability purposes. The same materials were used as in Experiment 1. The selection and order of the stimuli were randomly assigned to each participant across conditions. For all participants, objects were used.

#### Response measures, interobserver agreement, and procedural fidelity

The main dependent variable, as in Experiment 1, was percentage of correct responses. This was defined as in Experiment 1.

A second independent observer recorded data for 36% of listener and speaker trials during the test sessions for each participant. Interobserver reliability was measured as in Experiment 1. During test sessions, overall agreement during test sessions was 100% for listener trials and 88.9% for speaker trials for P4, was 94.4% for listener trials and 94.4% for speaker trials for P5, and 100% for listener trials and 94.4% for speaker trials for P6.

We assessed procedural fidelity for approximately 30% of sessions in the same manner as Experiment 1. Procedural fidelity for P4 was 93.9%, for P5 was 90.9%, and for P6 was 93.9%.

#### Procedure

Sessions were conducted in the same manner as Experiment 1.

##### Echoic pretest

This test preceded the study and was conducted in the same manner as Experiment 1.

##### General procedure

Each session involved one or more test administrations within the same phase. When a phase change occurred (i.e., from the with‐cues to the without‐cues condition, to the partial‐cues condition), we began a new session on a separate day. Sessions for all participants lasted on average between 50 and 70 min. It took six sessions for each participant. During each session, the experimenter administered naming tests, which involved an object and its name being presented to the child followed by a series of listener and speaker trials (all conducted nonsimultaneously). Each test was administered with a novel object and name and the naming experience was either conducted with, without, or with partial contextual cues (see description of the three conditions below).

##### Naming test with‐cues condition

This test was conducted in the same manner as described for Experiment 1.

##### Naming test without‐cues condition

This test was also conducted in the same manner as described for Experiment 1.

##### Naming test with‐partial‐cues condition

*Three object‐sound exposure trials*. The only difference between the with‐cues and with‐partial‐cues conditions was as follows: In the with‐partial‐cues condition, linguistic cues and gaze shift were removed. Specifically, when the child made visual contact with the novel object used during the session, the experimenter placed the object back in the box, left the box on the table, and said, “[the name of the object]” (e.g., “BOBO”). The experimenter pointed to the box but did not look at the object and kept her gaze focused away from the child and the box while saying the name of the object. There were no other planned differences between the two conditions.

*Intertrial intervals*. These were the same as the with‐ and without‐cues conditions for Experiment 1.

*Three speaker trials*. These were conducted similarly to those in the with‐cues condition, except the experimenter did not look at the object/box while asking, “What is this?”

*Three listener trials*. These were conducted similarly to those in the with‐cues condition, except that the experimenter gazed away from the child and the box when presenting the instruction to “Pick up” or “Point to” the previously presented object. The experimenter gazed toward the participant once the participant selected an object from the array.

Similar to the with‐ and without‐cues conditions, during each phase of the partial‐cues condition, we conducted three tests each with novel stimuli.

#### Experimental design and data analysis

An ABACBC reversal design was used wherein A was the with‐cues condition, B was the without‐cues condition, and C was the partial‐cues condition. Pearson's chi‐square analyses were used to compare participants' responses during the different testing phases (with‐cues, without‐cues, with‐partial‐cues).

### Results and Discussion

**FIGURE 2:**
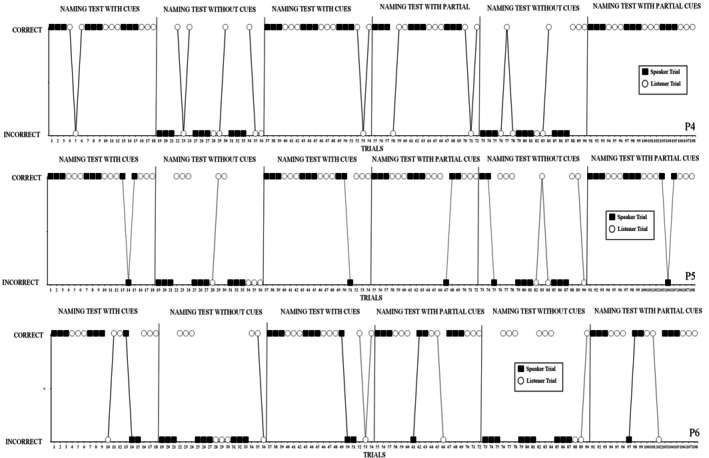
Responses of P4, P5, and P6 during administrations of the naming test with, without, and with partial cues.

The results of all three participants are displayed in Figure 2. P4 emitted 18/18 correct speaker responses and 16/18 correct listener responses during the with‐cues condition. Comparatively, P4 had 0/18 correct speaker responses and 9/18 correct listener responses in the without‐cues condition. Furthermore, P4 had 18/18 correct speaker responses and 16/18 correct listener responses in the partial‐cues condition. P5 produced 16/18 correct speaker responses and 18/18 correct listener responses in the with‐cues condition. Comparatively, P5 produced only 2/18 correct speaker responses and 11/18 correct listener responses in the without‐cues condition. Furthermore, P5 emitted 16/18 correct speaker responses and 18/18 correct listener responses in the partial‐cues condition. P6 demonstrated 15/18 correct speaker responses and 16/18 correct listener responses during the with‐cues condition. Comparatively, P6 had 0/18 correct speaker responses and 12/18 correct listener responses in the without‐cues condition. Furthermore, P6 emitted 16/18 correct speaker responses and 16/18 correct listener responses in the partial‐cues condition.

The results indicate that all participants produced overall more correct responses during test conditions with cues and with partial cues than during those without cues. The chi‐square test indicated that the total number of correct responses on listener and speaker trials was significantly associated with the presence or absence of all or partial cues, χ^2^_(1)_ = 137.82, *p* < .0001. More specifically, a participant was more likely to emit a correct speaker or listener response during trials where stimuli were presented with cues or with partial cues and unlikely to emit a correct speaker or listener response during trials presented without cues. Post hoc comparisons using α = .017, applying the Bonferroni correction, indicated that correct responses were (1) not significantly associated with the presence of all cues or a partial set of cues, χ^2^_(1)_ = 0.24, *p* = .06, (2) were significantly associated with the presence of no cues or a partial set of cues, χ^2^_(1)_ = 92.36, *p* < .0001, and (3) were significantly associated with the presence of no cues and all cues, χ^2^_(1)_ = 86.39, *p* < .0001.

Participants generally emitted no or relatively fewer correct speaker responses during the without‐cues condition than during the with‐cues and partial‐cues conditions. Participants were more likely to emit correct listener responses during the without‐cues condition, but these were still fewer than during the with‐cues and partial‐cues conditions. A chi‐square analysis indicated that responses during speaker trials were significantly associated with the presence or absence of all or some cues, χ^2^_(1)_ = 115.59, *p* < .0001. Post hoc comparisons using α = .017, applying the Bonferroni correction, indicated that correct speaker responses (a) were not significantly associated with the presence of all cues or a partial set of cues, χ^2^_(1)_ = 0.44, *p* = .50, (b) were significantly associated with the presence of no cues or a partial set of cues, χ^2^_(1)_ = 85.45, *p* < .0001, and (c) were significantly associated with the presence of no cues and all cues, χ^2^_(1)_ = 78.80, *p* < .0001.

A chi‐square analysis indicated that responses during listener trials were also significantly associated with the presence or absence of all or some cues, χ^2^_(1)_ = 182.91, *p* < .0001. Post hoc comparisons using α = .017, applying the Bonferroni correction, indicated that correct listener responses (a) were not significantly associated with the presence of all cues or a partial set of cues, χ^2^_(1)_ = 0, *p* = 1.00, (b) were significantly associated with the presence of no cues or a partial set of cues, χ^2^_(1)_ = 16.41, *p* < .0001, and (c) were significantly associated with the presence of no cues and all cues, χ^2^_(1)_ = 16.41, *p* < .0001.

Overall, the results of Experiment 2 demonstrate that autistic participants were more likely to emit correct naming responses during test conditions in which stimuli were presented with cues and with partial cues than during test conditions where stimuli were presented without cues. These findings further highlight the importance of specific cues, in this case pointing, during naming experiences.

The three experimental conditions differed not only in terms of the contextual cues presented during the naming experience (i.e., the three exposures to each object and its name) but also in terms of cues presented on test trials for speaker and listener relations. It could be argued that by not conducting test trials in the same manner across all conditions, differences between conditions could be due to the presence or absence of cues in the test trials (where cues could have served, for example, to promote *mere observation* of the stimuli and thereby facilitate correct responses). To address this, a third experiment was conducted in which only the exposure trials differed in terms of the presence of absence of cues and test trial presentation was consistent across conditions. If presenting contextual cues during the test per se were sufficient for naming, then there should be little or no difference when cues are manipulated during prior exposure trials.

## EXPERIMENT 3

### Method

#### Participants

The participants were three autistic children. At the beginning of the study, P7 was a 4‐year‐and‐11‐month‐old male, P8 was a 5‐year‐and‐11‐month‐old male, and P9 was a 6‐year‐and‐4‐month‐old female. All participants completed the study within 7 weeks of beginning the study. All participants had previously completed the core skills assessment described in Experiments 1 and 2 and were found to have the same preexisting skills. These participants, like those in Experiment 2, were noted to be unable to engage in extended conversations.

#### Setting and materials

The setting was the same as Experiment 1 and Experiment 2, and all sessions were again videotaped for reliability purposes. We used the same materials as in both Experiments.

#### Response measures, interobserver agreement and procedural fidelity

The main dependent variable was percentage of correct responses, which was measured in the same way as the prior two experiments. A second independent observer recorded data for 33.33% of listener and speaker trials during the test sessions for each participant, as in the first two experiments. For test sessions, overall agreement was 100% for listener trials and 93.3% for speaker trials for P7, 100% for listener trials and 86.6% for speaker trials for P8, and 93.3% for both listener and speaker trials for P9.

We assessed procedural fidelity for approximately 30% of sessions in the same manner as Experiments 1 and 2. Procedural fidelity for P7 was 100%, for P8 was 96.2%, and for P9 was 92.6%.

#### Procedure

Sessions were conducted in the same manner as Experiments 1 and 2.

##### Echoic pretest

This test preceded the study and was conducted in the same manner as Experiments 1 and 2.

##### General procedure

Each session involved one or more test administrations within the same phase. When a phase change occurred (i.e., from the with‐cues, to the without‐cues condition, to the partial‐cues condition), we began a new session on a separate day; in other words, each condition was completed in one session. Sessions for all participants lasted on average between 50 and 70 min. It took five sessions in total for each participant. During each session, the experimenter administered naming tests, which involved an object and its name being presented to the child followed by a series of listener and speaker trials (all conducted nonsimultaneously). Each test was administered with a novel object and name, and the naming experience was conducted either with or without contextual cues (see description of the two conditions below).

##### Naming test with‐cues condition

This test was conducted in the same manner as described for previous experiments.

##### Naming test without‐cues condition

This test was conducted in the same manner as described for Experiment 1.

#### Experimental design and data analysis

An ABABA/BABAB reversal design was used wherein A was the with‐cues condition and B was the without‐cues condition. We randomized the order in which participants underwent the two conditions. Participants 7 and 9 started with the with‐cues condition, and Participant 8 started with the without‐cues condition. Pearson's chi‐square analyses were used to compare participants' responses during the different testing phases (with cues and without cues).

### Results and discussion

**FIGURE 3:**
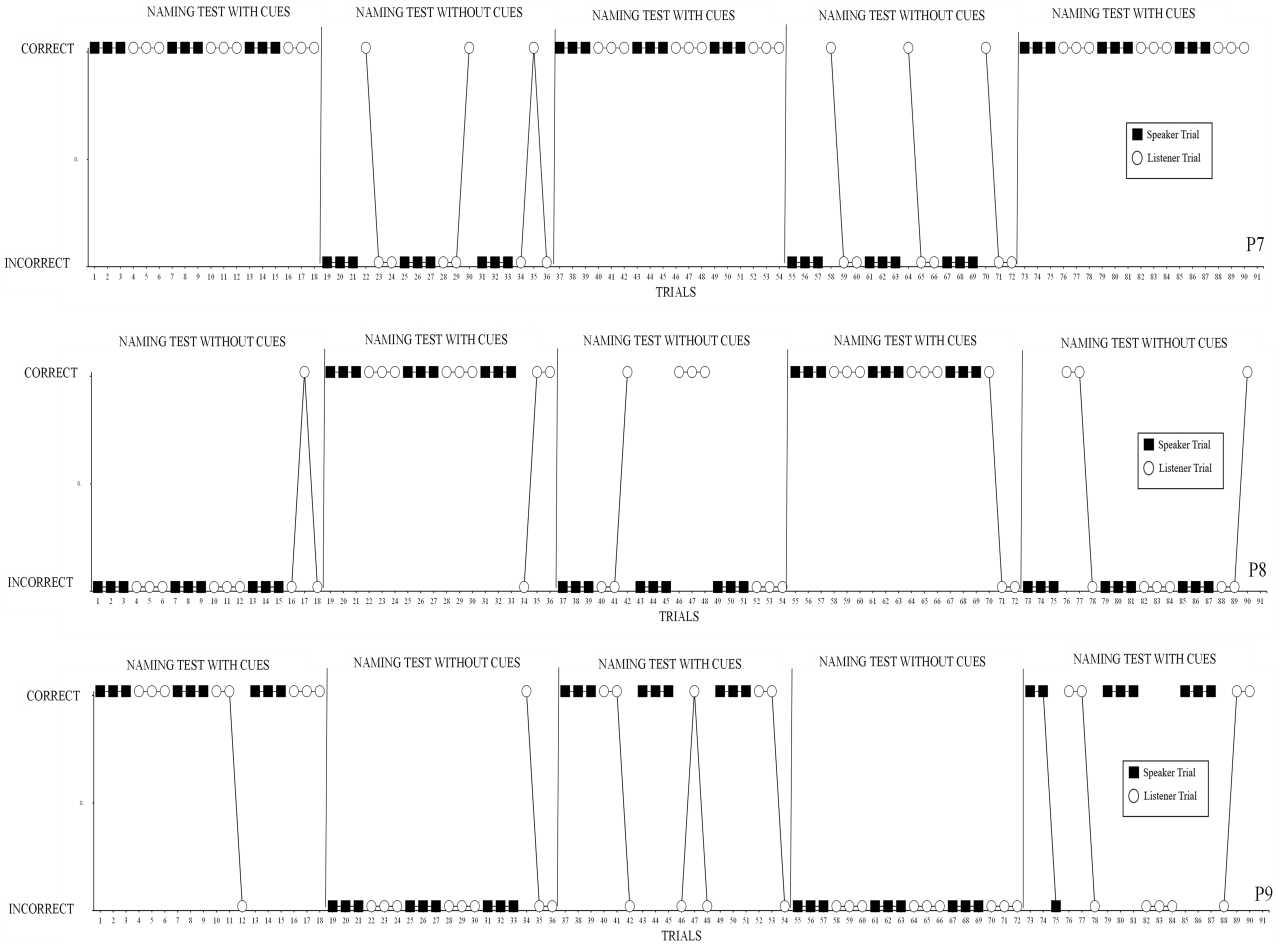
Responses of P7, P8, and P9 during administrations of the naming test with and without cues.

The results of all three participants are displayed in Figure 3. P7 emitted 27/27 correct speaker responses 27/27 correct listener responses during the with‐cues conditions. Comparatively, P7 had 0/18 correct speaker responses and had 6/18 correct listener responses in the with‐out cues condition. P8 produced 18/18 correct speaker responses and 15/18 correct listener responses in the with‐cues condition. Comparatively, P8 has 0/27 correct speaker responses and 5/27 correct listener responses in the without‐cues condition. P9 emitted 26/27 correct speaker responses and 17/27 correct listener responses during the with‐cues condition. Comparatively, P9 had 0/18 correct speaker responses and 1/18 correct listener responses in the without‐cues condition. These results indicate clear differences in naming when tests were presented with and without cues, with all participants producing more correct responses during test conditions with cues than during those without cues. A chi‐square test indicated that the total number of correct responses on listener and speaker trials was significantly associated with the presence or absence of cues, χ^2^_(1)_ = 175.76, *p* < .0001. More specifically, participants were more likely to emit a correct speaker or listener response during trials where stimuli were presented with cues and less likely to emit a correct speaker or listener response during trials presented without cues.

Participants generally emitted no or relatively few correct speaker responses during the without‐cues condition relative to the with‐cues condition. Furthermore, participants were more likely to emit correct listener responses during the without‐cues condition, but these were fewer than during the with‐cues condition. A chi‐square analysis indicated that responses during speaker trials was significantly associated with the presence or absence of cues, χ^2^_(1)_ = 131.04, *p* < .0001, and that responses during listener trials were also significantly associated with the presence or absence of cues, χ^2^_(1)_ = 53.31, *p* < .0001. Thus, although both speaker and listener responses were influenced by the presence or absence of cues, speaker responses appeared to be more so.

Experiment 3 examined the influence of three contextual cues on naming responses in autistic children. Participants consistently demonstrated higher accuracy in naming when stimuli had been previously presented with cues, despite identical test procedures across conditions. These findings provide evidence that the presence of contextual cues during exposure rather than during testing facilitates the acquisition of naming responses. These findings further support the important role of contextual cues in supporting early word learning in autistic children.

## GENERAL DISCUSSION

The current study was an initial attempt to investigate the role of contextual cues presented during a naming experience in facilitating listener and speaker naming responses. Across three experiments involving nine participants, we found that all children performed better on naming tests with contextual cues than those in which cues were not presented. Critically, our results highlight that when contextual cues (e.g., gaze shift between object and child, pointing and linguistic cues such as “*This is* [object name]”) are not presented, successful naming is undermined. These effects were observed specifically when temporal contiguity (a potential cue itself) between an object and its name was not present. Our findings show that contextual cues facilitate correct naming responses when they are presented during a naming experience. Furthermore, children emitted correct responses even with a partial set of cues, specifically, pointing toward the target stimulus. Given the current findings, it appears that specific contextual cues, such as pointing, facilitate “bridging the temporal gap” to support naming experiences during nonsimultaneous stimulus presentations. Although beyond the scope of the current study, future research might seek to explore the extent to which mediating vocal responses are involved in bridging this temporal gap. For example, perhaps participants were more likely to covertly echo the names of stimuli when cues were presented than when they were not (see Horne & Lowe, 1996).

In Experiment 1, specific cues were not systematically manipulated and thus we were not able to conclude whether some cues were perhaps more important than the other cues during a naming experience. In Experiment 2, along with attempting to replicate the findings of Experiment 1 with autistic participants, we also investigated the influence of pointing in facilitating naming. We found that pointing appeared to be a significant cue in facilitating naming experiences when other contextual cues (temporal contiguity, linguistic cues) were not present. Furthermore, the results of Experiment 2 provided additional support for the role of specific contextual cues in naming experiences, extending the findings to autistic children. As mentioned, some autistic children demonstrate difficulties in understanding the cues of others (e.g., Jellema et al., 2009), so the current findings have applied relevance for the use of such contextual cues (pointing, looking/orienting, linguistic) in that they can be targeted in training and/or testing for naming in this population. Finally, the results of Experiment 3 also demonstrated that presence of cues during the naming test alone were not sufficient to promote naming when those cues were absent during the prior exposure trials. The results of the current study are consistent with findings in mainstream psychology in highlighting the role of contextual cues in enhancing children's responses to new words (Lee & Lew‐Williams, 2022; Striano et al., 2006).

The current study presents several interesting findings that are worth further discussion. First, speaker responses were more adversely affected than listener responses in the absence of cues. Behavior analysts across theoretical perspectives have documented an asymmetry between listener and speaker naming responses in that listener naming responses seem to emerge more readily than speaker responses (e.g., Greer et al., 2005; Horne et al., 2006; Lowe et al., 2002; Miguel & Kobari‐Wright, 2013). Listener responses involve producing behaviors that are topographically similar to those that occur during a naming experience, whereas speaker responses involve behaviors that differ topographically from the original experience (i.e., the child may hear the name during the naming experience, but they mus vocally reproduce that name as a speaker). Therefore, speaker naming involves a response topography that is relatively more complex than the topography involved in listener naming (Sivaraman et al., 2021). Furthermore, in listener trials, children choose from an array of objects (e.g., three), so their selection might be influenced by chance, giving a one in three probability of correct selection. In contrast, speaker trials do not involve random choice and require the emission of an accurate verbal response without any options to choose from.

A related issue is that during the listener trials, it could be argued that the participants' object selections were controlled by familiarity of the object that was presented in the previous exposure and speaker trials rather than the auditory (nonsense name) stimulus. However, if familiarity was the main variable responsible for selecting the correct object during listener trials, one would expect participants to produce similar responding across all conditions, but this was not the case. Participants were more likely to emit a correct response during listener trials than during speaker trials in the without‐cues condition; however, they were less likely to emit correct responses that they were in the with‐cues condition. Furthermore, it is important to note that during the intertrial intervals (between exposure and test trials), the experimenter frequently engaged in unrelated conversation and play with the participants, thus stretching a simple familiarity‐based explanation for the current findings. Nevertheless, future studies could certainly include controls for stimulus exposure/attention.

As an aside, prior theoretical arguments have posited that echoic responses may be necessary for correct speaker naming responses (e.g., Horne & Lowe, 1996). Interestingly, all our participants emitted correct echoic responses to the chosen stimuli (i.e., they could all emit the motor responses necessary to produce the vocal sounds that were assigned to each novel object). Nevertheless, despite doing so, the removal of contextual cues was negatively associated with the number of correct speaker responses.

In Experiment 2, pointing alone significantly enhanced children's naming responses during the partial‐cue conditions, producing results similar to naming tests conducted with all cues. This aligns with prior research in developmental psychology wherein children as young as 7–10 months of age have been shown to communicate using gestures like pointing before acquiring verbal labels for objects (Butcher & Goldin‐Meadow, 2000; Capirci et al., 1996). Researchers have demonstrated that children use pointing, also called deictic gestures, to refer to an object that is physically present around them, and they also respond to their caregivers' pointing by orienting toward that direction (see Morford & Goldin‐Meadow, 1992, for a study on gesture comprehension in preverbal toddlers). In effect, children seem to orient in the direction of a caregiver's pointing prior to the development of responding in the direction of a caregiver's eye gaze (Mundy et al., 2007).

From a behavioral perspective, it is possible that pointing was so significant in supporting naming responses due to a long history of reinforcement tied to orienting in the general direction specified by pointing. Such paralinguistic cues are an integral part of one's verbal behavior and play important functions in controlling the behavior of the listener (Baldwin & Moses, 1994; Pelaez et al., 2012; Skinner 1957). For example, looking in the direction of a caregiver's pointing finger may often lead to interesting or valuable visual consequences for a child from early on in their life. An important step for future research would be to systematically vary pointing and orienting. For example, it may be useful to investigate the influence of orienting back‐and‐forth between the target object and the child while pointing toward the ceiling or some other irrelevant direction (i.e., thereby creating a tension between two paralinguistic cues).

As mentioned, the current study reintroduced only one cue (pointing) in the second experiment. Therefore, future research should investigate the roles of linguistic cues (e.g., “This is …”) and eye gaze by systematically removing and reintroducing them across various conditions. Indeed, partial cues were always implemented following the participant's exposure to two phases in which all cues were present. Without this prior exposure, perhaps the partial cues would not have been as effective; a future study could address this issue. Furthermore, it may be that with younger children a combination of cues is needed to reestablish naming, and such young children may be less likely to demonstrate naming even with temporal contiguity alone removed, as reported by Sivaraman et al. (2021). These researchers found that toddlers did not emit correct listener or speaker responses when the object and its name were shown nonsimultaneously (omitting a critical paralinguistic cue, i.e., holding the object while naming it), but after training they responded correctly, suggesting that additional cues (e.g., pointing and stating, “that is a …”) began to control the toddlers' responses following the training. As children grow older, however, linguistic cues alone may come to control their naming responses. Future research may also examine the influence of the nonsimultaneous presentation format, combined with the manipulation of cues, on older children and adults. It is presumed that due to their extensive verbal behavior and reinforcement histories, there would be little effect of such stimuli with adults. Nonetheless, it may be important to test such an assumption to allow for comparisons with naming abilities of children during similar tasks.

The present findings certainly have applied relevance. In the natural environment it is very likely that children are learning the names of objects via nonsimultaneous presentations. For example, imagine a parent and a child are watching a video and a dinosaur appears on the screen. Following watching the video the parent may ask the child, “Did you see the dinosaur just then?” Therefore, it is important to systematically investigate naming under these types of circumstances. At present, only one published study (Sivaraman et al., 2021) has examined naming when the names and objects were presented nonsimultaneously. In their study, however, the potential role of specific contextual cues presented during naming experiences was not explored. The present study marks a first step in exploring the role that such contextual cues play on naming responses in children.

A limitation to note within the current study was the use of a table‐top instructional setting whereby sessions took place in a research room. In natural settings, children often come across new objects or pictures when caregivers introduce these stimuli during activities like play, reading, or social interactions (Hart & Risley, 1995, 1999). Although some prior research on naming has explored such naturalistic contexts (e.g., Carey & Bartlett, 1978), there remains a need for further studies on how children encounter and learn new words in everyday situations. Ecologically valid research that reflects real‐life scenarios where children learn and use naming responses is crucial for understanding verbal behavior development. For example, a future study might incorporate nonsimultaneous presentations through a storybook where a picture pops up and the name appears on a subsequent page; a relevant cue might then be manipulated by the reader pointing to, or not pointing to, the pop‐up picture. In any case, although the present study was conducted in a less naturalistic setting, it incorporated incidental naming encounters where errors were not corrected, and correct responses were not reinforced with social praise. Additionally, toys and activities were within the children's reach during sessions, allowing them to shift their attention away from the experimenter and return to these activities, somewhat mirroring the natural environment.

Another limitation to note in Experiments 1 and 2 is that all participants began with naming tests with cues before moving to other conditions. This sequential approach, combined with the lack of randomization in the order of conditions, potentially introduced a systematic bias. Without randomizing the starting phase, we cannot rule out the possibility that the initial exposure to cues influenced participants' responses in subsequent conditions. Experiment 3 did incorporate some aspect of randomization by beginning with a without‐cues condition as opposed to a with‐cues condition, and it appeared that the effects observed were not affected by the order of presentation of conditions. Furthermore, we ran each condition on different days to reduce any carryover effects.

To conclude, the current study highlights the critical role that contextual cues (e.g., linguistic and paralinguistic) play in facilitating naming responses, particularly when temporal contiguity is absent during the presentation of a novel object and its name. We used the framework offered by RFT to describe and manipulate contextual cues during a naming experience, thereby deepening our understanding of naming. This approach not only advances the study of naming but also enriches the science of behavior analysis by considering diverse theoretical perspectives in an empirical investigation on naming. The experiments demonstrated that although paralinguistic and linguistic cues significantly aided naming responses, future research should focus on the manipulation of individual cues and their combinations across various contexts and populations. Indeed, language acquisition outside of the laboratory and highly structured teaching settings does include many nuances of the linguistic interaction that have not been systematically addressed in prior research, so the current study offers a first step in systematically evaluating their efficacy. Overall, the current study highlights how an intricate understanding of contextual cues presented during a naming experience is fundamental to our understanding of the variables that facilitate naming in both typically developing and autistic children.

## AUTHOR CONTRIBUTIONS

NH, MS, DBH contributed equally to conceptualizing the research idea, designing the study, and interpreting the data. NH generated the experimental tasks and collected the data. NH wrote an initial draft of the manuscript, and all authors worked equally on the manuscript thereafter and approved the final version.

## CONFLICT OF INTEREST STATEMENT

The authors declare no conflicts of interest.

## ETHICS APPROVAL

Ethical approval for all experiments was obtained from the first author's educational institution where she is enrolled as a doctoral student and from the center in which she worked. Parents of the participants provided written informed consent, and verbal assent was sought from each participant prior to each session.

## Supporting information


**Data S1.** Supporting Information

## Data Availability

The data that support the findings of this study are available from the corresponding author upon reasonable request.

## Footnotes

^1^
Indeed, studying the influence of delays may be directly relevant to the concept of displacement, which refers to referential language in the absence of the thing being talked about (Hockett, 1960; Skinner, 1953).

## References

[jeab70059-bib-0043] Anderson, D. K. , Lord, C. , Risi, S. , DiLavore, P. S. , Shulman, C. , Thurm, A. , Welch, K. , & Pickles, A. (2007). Patterns of growth in verbal abilities among children with autism spectrum disorder. Journal of consulting and clinical psychology, 75(4), 594–604. 10.1037/0022-006X.75.4.594 17663613

[jeab70059-bib-0001] Akhtar, N. , Jipson, J. , & Callanan, M. A. (2001). Learning words through overhearing. Child Development, 72(2), 416–430. 10.1111/1467-8624.00287 11333075

[jeab70059-bib-0002] Baldwin, D. A. , & Moses, L. J. (1994). Early understanding of referential intent and attentional focus: Evidence from language and emotion. In C. Lewis & P. Mitchell (Eds.), Children's early understanding of mind: Origins and development (pp. 133–156). Lawrence Erlbaum Associates.

[jeab70059-bib-0003] Bates, E. , Camaioni, L. , & Volterra, V. (1975). The acquisition of performatives prior to speech. Merrill‐Palmer Quarterly of Behavior and Development, 21(3), 205–226.

[jeab70059-bib-0004] Bertenthal, B. I. , Boyer, T. W. , & Harding, S. (2014). When do infants begin to follow a point? Developmental Psychology, 50(8), 2036–2048. 10.1037/a0037152 24911570

[jeab70059-bib-0005] Butcher, C. , & Goldin‐Meadow, S. (2000). Gesture and the transition from one‐to two‐word speech: When hand and mouth come together. In D. McNeill (Ed.), Language and gesture (pp. 235–258). Cambridge University Press.

[jeab70059-bib-0006] Butterworth, G. (2003). Pointing is the royal road to language for babies. In S. Kita (Ed.), Pointing: Where language, culture, and cognition meet (pp. 9–33). Lawrence Erlbaum Associates Publishers. 10.1037/a0037152

[jeab70059-bib-0007] Capirci, O. , Iverson, J. M. , Pizzuto, E. , & Volterra, V. (1996). Gestures and words during the transition to two‐word speech. Journal of Child language, 23(3), 645–673. 10.1017/S0305000900008989

[jeab70059-bib-0008] Carey, S. , & Bartlett, E. (1978). Acquiring a single new word. Proceedings of the Stanford Child Language Conference, 15, 17–29.

[jeab70059-bib-0009] Carpenter, M. , Nagell, K. , Tomasello, M. , Butterworth, G. , & Moore, C. (1998). Social cognition, joint attention, and communicative competence from 9 to 15 months of age. Monographs of the Society for Research in Child Development, 63(4), Publication 1166214. 10.2307/1166214 9835078

[jeab70059-bib-0010] de Villiers Rader, N. , & Zukow‐Goldring, P. (2012). Caregivers' gestures direct infant attention during early word learning: The importance of dynamic synchrony. Language Sciences, 34(5), 559–568. 10.1016/j.langsci.2012.03.011

[jeab70059-bib-0011] Dickson, C. A. , MacDonald, R. P. F. , Mansfield, R. , Guilhardi, P. , Johnson, C. , & Ahearn, W. H. (2014). Social validation of the New England Center for Children—Core Skills Assessment. Journal of Autism and Developmental Disorders, 44(1), 65–74. 10.1007/s10803-013-1852-5 23719854

[jeab70059-bib-0012] Fitch, A. , Lieberman, A. M. , Luyster, R. J. , & Arunachalam, S. (2020). Toddlers' word learning through overhearing: Others' attention matters. Journal of Experimental Child Psychology, 193, Article 104793. 10.1016/j.jecp.2019.104793 PMC7114829 31992441

[jeab70059-bib-0013] Gilic, L. , & Greer, R. D. (2011). Establishing naming in typically developing two‐year‐old children as a function of multiple exemplar speaker and listener experiences. Analysis of Verbal Behavior, 27(1), 157–177. 10.1007/BF03393099 22532761 PMC3139556

[jeab70059-bib-0014] Gilmore, A. , Barnes‐Holmes, D. , & Sivaraman, M. (2024). A modern collaborative behavior analytic approach to incidental naming. Perspectives on Behavior Science, 47(3), 581–601. 10.1007/s40614-024-00399-0 39309234 PMC11411027

[jeab70059-bib-0015] Greer, R. D. , Corwin, A. , & Buttigieg, S. (2011). The effects of the verbal developmental capability of bidirectional naming on how children can be taught. Acta de Investigación Psicológica, 1(1), 23–54. 10.22201/fpsi.20074719e.2011.1.214

[jeab70059-bib-0016] Greer, R. D. , & Longano, J. (2010). A rose by naming: how we may learn how to do it. Analysis of Verbal Behavior, 26(1), 73–106. 10.1007/BF03393085 22477465 PMC2900947

[jeab70059-bib-0017] Greer, R. D. , Stolf, L. , Chavez‐Brown, M. , & Rivera‐Valdes, C. (2005). The emergence of the listener to speaker component of naming in children as a function of multiple exemplar instruction. Analysis of Verbal Behavior, 21(1), 123–134. 10.1007/BF03393014 22477318 PMC2774093

[jeab70059-bib-0018] Hart, B. , & Risley, T. R. (1995). Meaningful differences in the everyday experience of young American children. Paul Brookes.

[jeab70059-bib-0019] Hart, B. , & Risley, T. R. (1999). The social world of children learning to talk. Paul Brookes.

[jeab70059-bib-0020] Hawkins, E. , Gautreaux, G. , & Chiesa, M. (2018). Deconstructing common bidirectional naming: A proposed classification framework. Analysis of Verbal Behavior, 34(1–2), 44–61. 10. 1007/s40616‐018‐0100‐7 31976214 10.1007/s40616-018-0100-7 PMC6702485

[jeab70059-bib-0021] Hockett, C. F. (1960). The origin of speech. Scientific American, 203(3), 88–96. 14402211

[jeab70059-bib-0022] Horne, P. J. , Hughes, J. C. , & Lowe, C. F. (2006). Naming and categorization in young children: IV: Listener behavior training and transfer of function. Journal of the Experimental Analysis of Behavior, 85(2), 247–273. 10.1901/jeab.2006.125-04 16673828 PMC1472628

[jeab70059-bib-0023] Horne, P. J. , & Lowe, C. F. (1996). On the origins of naming and other symbolic behavior. Journal of the Experimental Analysis of behavior, 65(1), 185–241. 10.1901/jeab.1996.65-185 16812780 PMC1350072

[jeab70059-bib-0024] Jellema, T. , Lorteije, J. , van Rijn, S. , van t'Wout, M., de Haan, E. , van Engeland, H. , & Kemner, C . (2009). Involuntary interpretation of social cues is compromised in autism spectrum disorders. Autism Research, 2(4), 192–204. 10.1002/aur.83 19642087

[jeab70059-bib-0025] Lee, C. , & Lew‐Williams, C. (2022). Speech and social cues combine at discourse boundaries to promote word learning. Cognitive Development, 64, Article 101254. 10.1016/j.cogdev.2022.101254

[jeab70059-bib-0026] Lowe, C. F. , Horne, P. J. , Harris, F. D. A. , & Randle, V. R. L. (2002). Naming and categorization in young children: Vocal tact training. Journal of the Experimental Analysis of Behavior, 78(3), 527–549 12507018 10.1901/jeab.2002.78-527 PMC1284914

[jeab70059-bib-0027] Miguel, C. F. (2016). Common and intraverbal bidirectional naming. The Analysis of Verbal Behavior, 32(2), 125–138. 10.1007/s40616-016-0066-2 30800621 PMC6381345

[jeab70059-bib-0028] Miguel, C. F. , & Kobari‐Wright, V. V. (2013). The effects of tact training on the emergence of categorization and listener behavior in children with autism. Journal of Applied Behavior Analysis, 46(3), 669–673. 10.1002/jaba.62 24114230

[jeab70059-bib-0029] Morford, M. , & Goldin‐Meadow, S. (1992). Comprehension and production of gesture in combination with speech in one‐word speakers. Journal of Child Language, 19(3), 559–580. 10.1017/S0305000900011569 1429948

[jeab70059-bib-0030] Mundy, P. , Block, J. , Delgado, C. , Pomares, Y. , Van Hecke, A. V. , & Parlade, M. V. (2007). Individual differences and the development of joint attention in infancy. Child Development, 78(3), 938–954. 10.1111/j.1467-8624.2007.01042.x 17517014 PMC2654237

[jeab70059-bib-0031] Olaff, H. S. , & Holth, P. (2020). The emergence of bidirectional naming through sequential operant instruction following the establishment of conditioned social reinforcers. The Analysis of Verbal Behavior, 36(1), 21–48. 10.1007/s40616-019-00122-0 32699737 PMC7343676

[jeab70059-bib-0032] Pelaez, M. , Virues‐Ortega, J. , & Gewirtz, J. L. (2012). Acquisition of social referencing via discrimination training in infants. Journal of Applied Behavior Analysis, 45(1), 23–36. 10.1901/jaba.2012.45-23 22403447 PMC3297351

[jeab70059-bib-0033] Regaço, A. , Harte, C. , Barnes‐Holmes, D. , Leslie, J. , & de Rose, J. C. (2025). Naming, stimulus equivalence and relational frame theory: Stronger together than apart. Perspectives on Behavior Science, 48(1), 97–114. 10.1007/s40614-024-00427-z 40078355 PMC11893944

[jeab70059-bib-0034] Rowe, M. L. (2000). Pointing and talk by low‐income mothers and their 14‐month‐old children. First Language, 20(60), 305–330. 10.1177/014272370002006005

[jeab70059-bib-0035] Scofield, J. , & Behrend, D. A. (2011). Clarifying the role of joint attention in early word learning. First Language, 31(3), 326–341. 10.1177/0142723710395423

[jeab70059-bib-0036] Sivaraman, M. , Barnes‐Holmes, D. , & Roeyers, H. (2021). Nonsimultaneous stimulus presentations and their role in listener naming. Journal of the Experimental Analysis of Behavior, 116(3), 300–313. 10.1002/jeab.715 34542178

[jeab70059-bib-0037] Sivaraman, M. , & Barnes‐Holmes, D. (2023). Naming: What do we know so far? A systematic review. Perspectives on Behavior Science, 46(3), 585–615. 10.1007/s40614-023-00374-1 38144546 PMC10733260

[jeab70059-bib-0038] Skinner, B. F. (1953). Science and human behavior. Macmillan.

[jeab70059-bib-0039] Skinner, B. F. (1957). Verbal behavior. Appleton‐Century‐Crofts. 10.1037/11256-000

[jeab70059-bib-0040] Slaughter, V. , & McConnell, D. (2003). Emergence of joint attention: Relationships between gaze following, social referencing, imitation, and naming in infancy. The Journal of Genetic Psychology, 164(1), 54–71. 10.1080/00221320309597503 12693744

[jeab70059-bib-0041] Striano, T. , Chen, X. , Cleveland, A. , & Bradshaw, S. (2006). Joint attention social cues influence infant learning. European Journal of Developmental Psychology, 3(3), 289–299. 10.1080/17405620600879779

[jeab70059-bib-0042] Tenenbaum, E. J. , Amso, D. , Abar, B. , & Sheinkopf, S. J. (2014). Attention and word learning in autistic, language delayed and typically developing children. Frontiers in Psychology, 5, Article 490. 10.3389/fpsyg.2014.00490 PMC4033261 24904503

