# Supplementary material for: Three contextual cues and their influence on naming in children
Source: J Exp Anal Behav. 2025 Oct 10;124(3):e70059. doi: 10.1002/jeab.70059 (PMC12513545; doi:10.1002/jeab.70059)
Supplement: Supplementary file 1 — Data S1. Supporting Information [file JEAB-124-0-s001.docx]

**Table 3.1** *Stimuli used During Training for Experiments 1, 2 and 3*

| Stimulus | Name | Picture |
| --- | --- | --- |
| 1 | MESA | 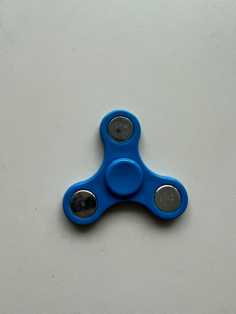 |
| 2 | BOBO | 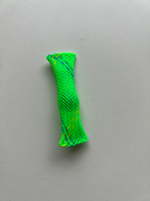 |
| 3 | NAJ | 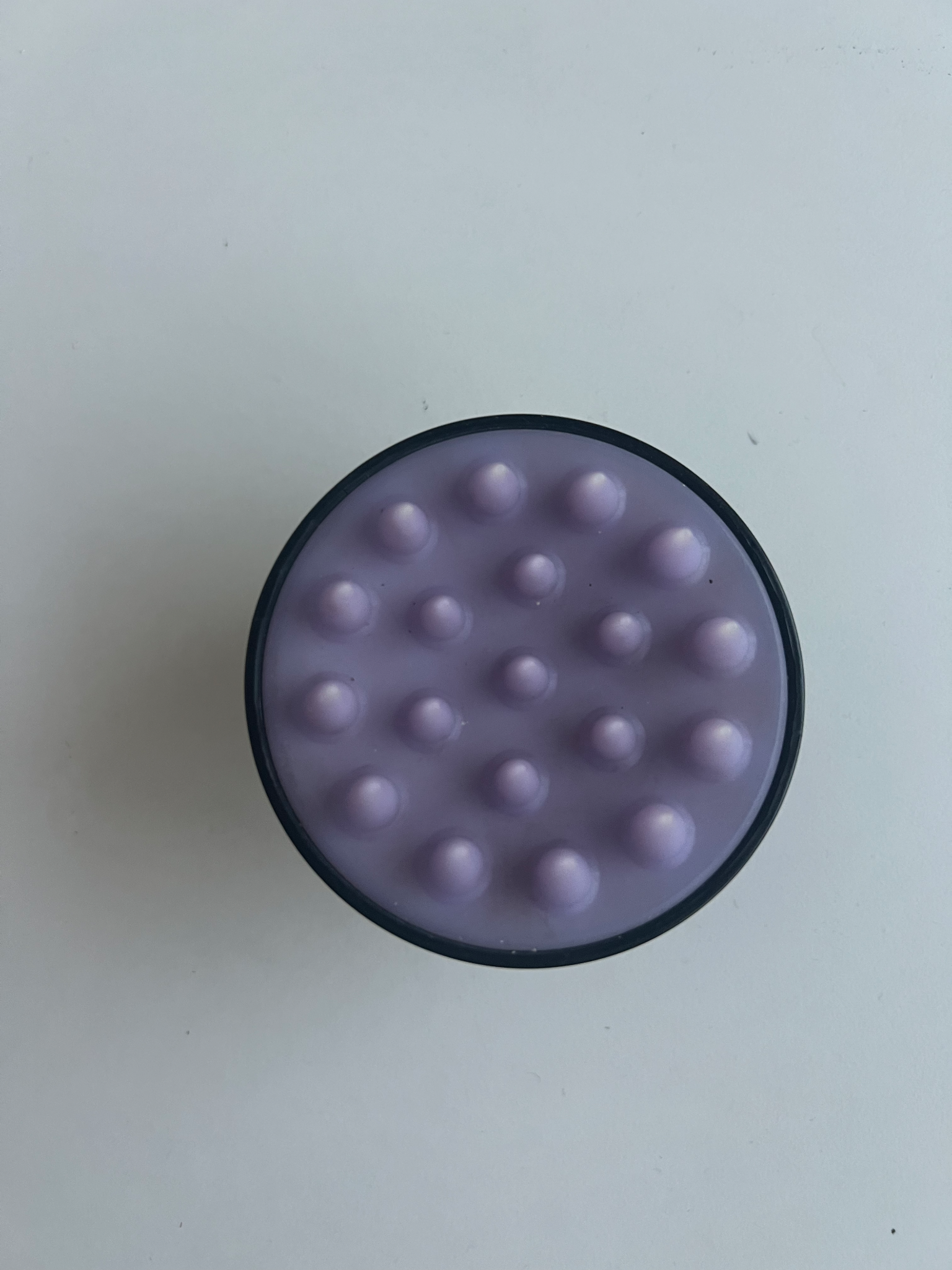 |
| 4 | BEK | 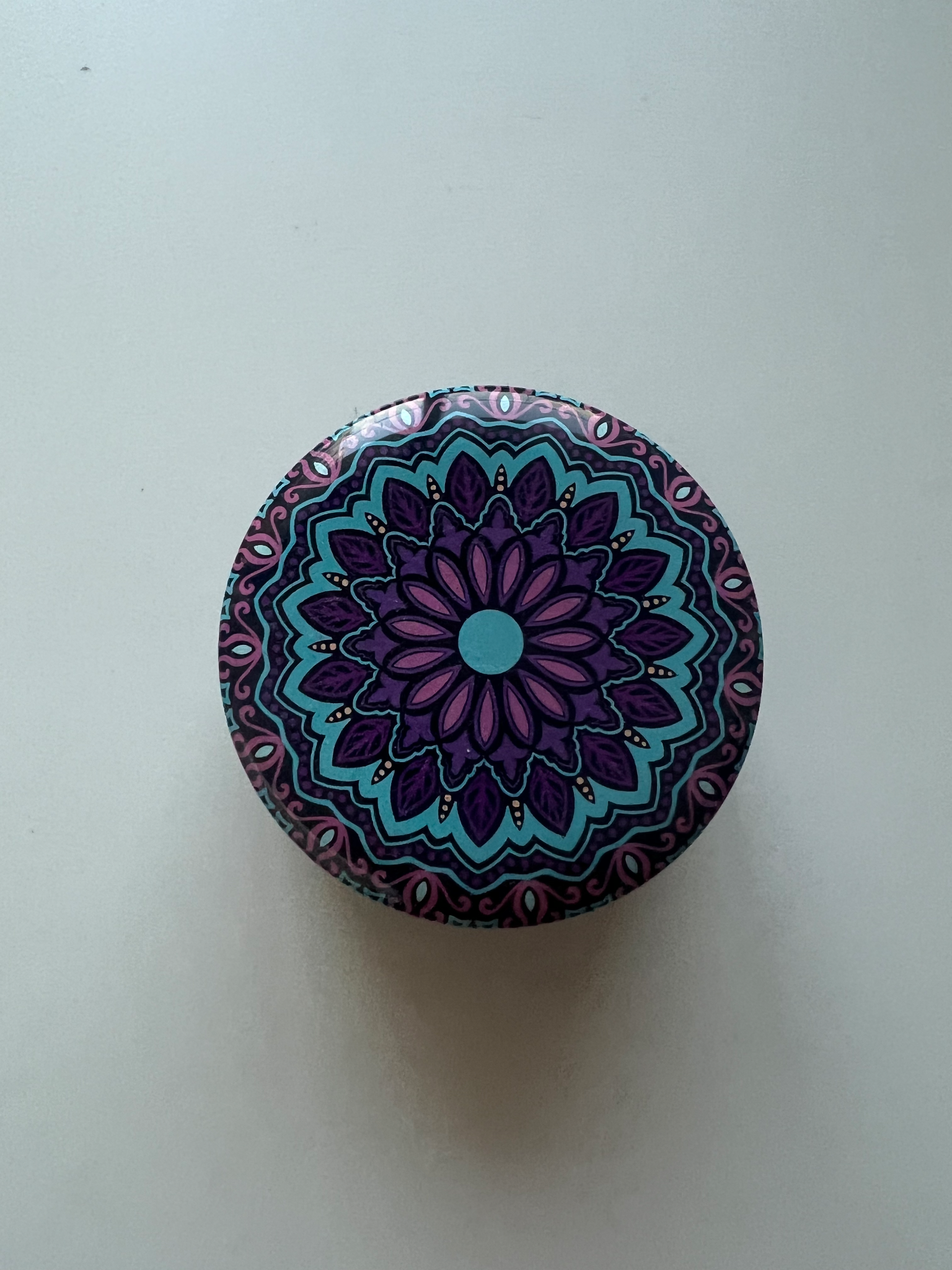 |
| 5 | FED | 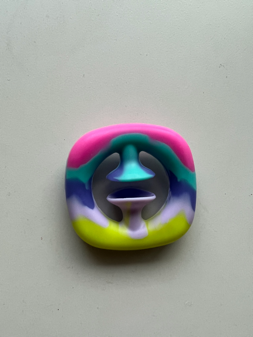 |
| 6 | AHMIT | 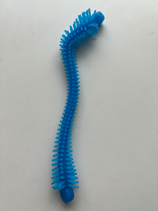 |
| 7 | MOT | 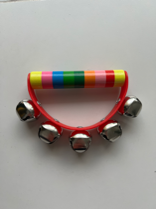 |
| 8 | TIC | 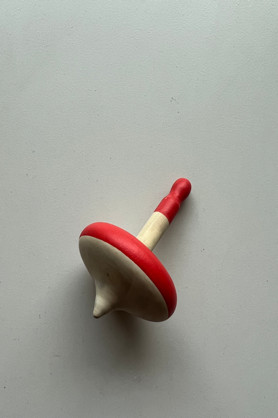 |
| 9 | PAM | 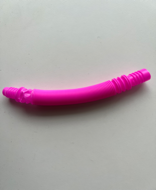 |
| 10 | ADD | 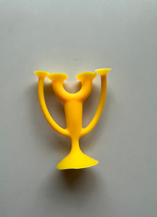 |
| 11 | TAM | 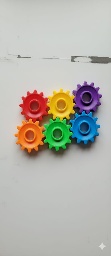 |
| 12 | COCO | 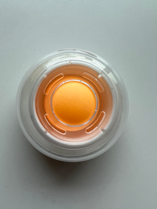 |
| 13 | VARK | 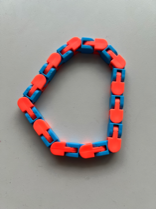 |
| 14 | DEL | 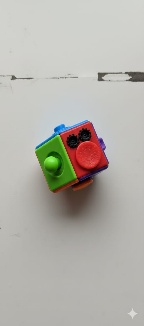 |
| 15 | JAD | 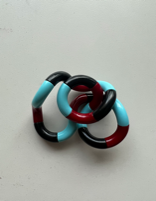 |
| 16 | SAP | 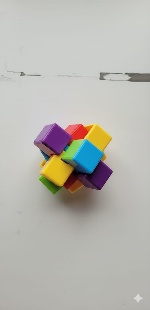 |
| 17 | ZAGGY | 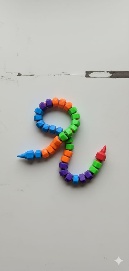 |
| 18 | TONO | 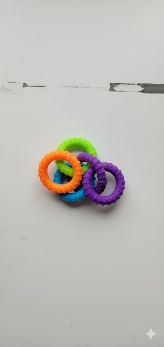 |

*Note.* Photos depict the physical objects used in the experiment. For P2, these photos were presented as stimuli in place of the physical objects.
